# Increased Inflammation in Atherosclerotic Lesions of Diabetic *Akita-LDLr* ^−/−^ Mice Compared to Nondiabetic *LDLr* ^−/−^ Mice

**DOI:** 10.1155/2012/176162

**Published:** 2012-11-28

**Authors:** Daniel Engelbertsen, Fong To, Pontus Dunér, Olga Kotova, Ingrid Söderberg, Ragnar Alm, Maria F. Gomez, Jan Nilsson, Eva Bengtsson

**Affiliations:** Department of Clinical Sciences Malmö, Skåne University Hospital, Lund University, CRC Entrance 72, 205 02 Malmö, Sweden

## Abstract

*Background*. Diabetes is associated with increased cardiovascular disease, but the underlying cellular and molecular mechanisms are poorly understood. One proposed mechanism is that diabetes aggravates atherosclerosis by enhancing plaque inflammation. The *Akita* mouse has recently been adopted as a relevant model for microvascular complications of diabetes. Here we investigate the development of atherosclerosis and inflammation in vessels of *Akita* mice on *LDLr* ^−/−^ background. *Methods and Results*. *Akita-LDLr* ^−/−^ and *LDLr* ^−/−^ mice were fed high-fat diet from 6 to 24 weeks of age. Blood glucose levels were higher in both male and female *Akita-LDLr* ^−/−^ mice (137% and 70%, resp.). Male *Akita-LDLr* ^−/−^ mice had markedly increased plasma cholesterol and triglyceride levels, a three-fold increase in atherosclerosis, and enhanced accumulation of macrophages and T-cells in plaques. In contrast, female *Akita-LDLr* ^−/−^ mice demonstrated a modest 29% increase in plasma cholesterol and no significant increase in triglycerides, atherosclerosis, or inflammatory cells in lesions. Male *Akita-LDLr* ^−/−^ mice had increased levels of plasma IL-1**β** compared to nondiabetic mice, whereas no such difference was seen between female diabetic and nondiabetic mice. *Conclusion*. *Akita-LDLr* ^−/−^ mice display considerable gender differences in the development of diabetic atherosclerosis. In addition, the increased atherosclerosis in male *Akita-LDLr* ^−/−^ mice is associated with an increase in inflammatory cells in lesions.

## 1. Introduction

Atherosclerosis is a chronic inflammatory disease characterized by formation of lesions in large- and medium-sized arteries. Diabetes is associated with increased atherosclerosis, and diabetic patients have a 2–4-fold increased risk of cardiovascular mortality [1, 2]. Moreover, stroke, coronary heart disease, and peripheral artery disease are more common and occur at an earlier age in diabetic patients than in nondiabetic persons [3]. The proposed culprits responsible for the increased risk of atherosclerosis in diabetic patients include dyslipidemia, hypertension, endothelial dysfunction, oxidative stress, and increased generation of advanced glycation end-products (AGEs) [4]. However, the underlying cellular and molecular mechanisms whereby diabetes accelerates atherosclerosis are still poorly understood, and one of the main reasons for this has been the lack of animal models of diabetes that replicate the disease as seen in humans.

One of the most widely used mouse models for cardiovascular disease in type 1 diabetes is the streptozotocin-induced diabetes model in atherosclerotic apolipoprotein E-deficient (*ApoE* ^−/−^) or LDL-receptor-deficient (*LDLr* ^−/−^) mice. The streptozotocin induced diabetes model, however, has a drawback in the nonspecific toxic effects of streptozotocin to other organs than pancreas, since streptozotocin is known to be both hepatotoxic and nephrotoxic [5]. In addition, streptozotocin can methylate DNA and has genotoxic effects by damaging DNA [6]. Recently, streptozotocin was shown to have a direct toxic effect on lymphocytes *in vitro*, particularly on CD8-cells and B-cells [7]. Streptozotocin treatment of mice also resulted in a relative increase in regulatory T-cells, and this effect was independent of hyperglycemia [7]. CD8-cells [8, 9], B-cells [10–13], and regulatory T-cells [14] have been shown to play important roles in atherosclerosis. Thus, these side effects of streptozotocin could have an impact on the atherosclerotic disease, which is not related to the diabetic disease. 

The *ins*2^*akita*/+^ *(Akita)* mouse is a model of type 1 diabetes, characterized by a point-mutation causing pro-insulin misfolding with subsequent endoplasmic reticulum-stress leading to beta-cell apoptosis [15]. The *Akita* mouse has previously been successfully used as a model of diabetic microvascular complications, including retinopathy, neuropathy, and nephropathy [16]. Recently, increased atherosclerosis in *Akita*-*Apo*-*E* ^−/−^ mice and *Akita*-*LDLr* ^−/−^ mice compared to nondiabetic mice has been reported [17, 18]. Both of these studies primarily focused on the role of altered lipid metabolism. Although the cellular and molecular mechanisms behind why diabetes results in increased atherosclerosis are not known, one proposed mechanism is increased inflammation, due to increased oxidative stress in the atherosclerotic lesions. In atherosclerosis, subendothelial retention and oxidation of LDL induce expression of proinflammatory cytokines and recruitment of inflammatory cells to the vessel wall [19, 20]. In diabetes, increased glucose levels result in formation of AGE, which is believed to increase oxidative stress and inflammation [21, 22]. To see if the increased atherosclerosis reported in *Akita*-*LDLr* ^−/−^ mice is accompanied or driven by increased plaque inflammation, we measured the extent of inflammatory cells and cytokines in atherosclerotic vessels of *Akita*-*LDLr* ^−/−^ mice. We also asked whether *Akita*-*LDLr* ^−/−^ mice display an altered immunological T-cell profile compared to nondiabetic *LDLr* ^−/−^ mice. 

## 2. Methods

### 2.1. Mice

*Ak* *it* *a*-*LDLr* ^−/−^ (B6.Cg-*Ins*2^*Akita*^ *LDLr* ^*tm*1*Her*^/J) and *LDLr* ^−/−^ mice were obtained from Jackson Laboratories and bred at our animal facilities. *Akita* mice were genotyped according to protocols provided by the Jackson Laboratories. Animals had free access to tap water and were fed a high-fat diet, containing 21% cocoa fat (weight%), 0.15% cholesterol (weight%), from six weeks of age, and were sacrificed at 18 or 24 weeks of age. Blood glucose was measured every three weeks using a One-Touch Glucometer (LifeScan Inc., CA, USA) in nonfasting mice. Plasma triglycerides and cholesterol were measured in nonfasting mice by colorimetric assays as described before [23]. All animal experiments were approved by the Malmö-Lund Animal Care and Use Committee and the investigation conforms to the Guide for the Care and Use of Laboratory Animals published by the United States National Institutes of Health.

### 2.2. Immunohistochemistry

For assessment of atherosclerosis, plaque characteristics, myocardial fibrosis, and inflammation mice were sacrificed at 24 weeks of age. *En face* preparations of the aorta, Oil red O-staining, and quantification were performed as described before [23]. Staining of monocytes/macrophages (MOMA-2, Biomedicals AG, Switzerland, detecting a glycoprotein located in the cytoplasm and on the cell surface on monocytes and macrophages), T-cells (anti-CD3) [24], collagen (Masson's trichrome), and AbcA1 (abcam) were performed as described before [23]. Subvalvular plaque area was determined in haematoxylin stained sections of the aortic root. 

### 2.3. Plasma Cytokines

Cytokines in plasma from 24-week-old mice were measured using a Th1/Th2 9-plex assay (Meso Scale Discovery, USA) according to manufacturer's instructions. 

### 2.4. Gene Expression Analysis

A separate subset of mice was sacrificed at 18 weeks of age to measure mRNA levels of inflammatory markers in brachiocephalic arteries. Mice were perfused with RNAlater (Applied Biosystems). Brachiocephalic arteries were isolated and snap-frozen in Trizol (Invitrogen). Total RNA was extracted as described previously [25] and cDNA was synthesized with RevertAid First Strand cDNA Synthesis Kit (Fermentas Life Sciences). mRNA levels were analyzed by quantitative real-time PCR using Taqman assays (Applied Biosystems): Mm00436767_m1 for osteopontin (OPN), Mm00446190_m1 for interleukin-6 (IL-6), Mm01336189_m1 for interleukin 1*β* (IL-1*β*), Mm01320970_m1 for vascular cell adhesion molecule (VCAM), Mm00442991_m1 for matrix metalloproteinase-9 (MMP9), Mm00436450_m1 for macrophage inflammatory protein-2 (MIP2), and Mm00441242_m1 for monocyte chemotactic protein-1 (MCP-1). Expression levels of target genes were normalized to the expression of cyclophilin B (PPIb), Mm00478295_m1, as the housekeeping gene. 

### 2.5. Splenocyte Preparation and Culture

Splenocytes were isolated as previously described [24]. Briefly, splenocytes from 24-week-old mice were isolated, washed, and stimulated with Concanavalin A (2.5 *μ*g/ml) or left unstimulated. After 72 hours, [methyl-^3^H]-thymidine was added to wells. To quantify DNA synthesis, cells were harvested 16 hours after [methyl-^3^H]-thymidine addition and measured using a liquid scintillation counter. 

### 2.6. Flow Cytometry

Splenocytes were washed and stained with fluorochrome-conjugated antibodies and analyzed with a CyAn ADP flow cytometer (Beckman Coulter). The antibodies used were AF488-CD69, PE-Cy7-CD3*ε*, PB-CD4, and APC-CD25 (all from Biolegend). 

### 2.7. Statistical Analysis

Values are presented as mean ± SD unless otherwise specified. Statistical analyses were performed with Graph-Pad 5 (Prism) or PASW Statistics 18 software. Statistical significance was determined using two-way ANOVA followed by Bonferroni post hoc tests unless otherwise specified. Correlation analyses were performed using Pearson (normally distributed variables) or Spearman (skewed variables). Linear regression analysis was performed with PASW Statistics 18.

## 3. Results

### 3.1. *Akita-LDL* ^−/−^ Mice Display Gender-Specific Metabolic Profiles

**Figure 1:**
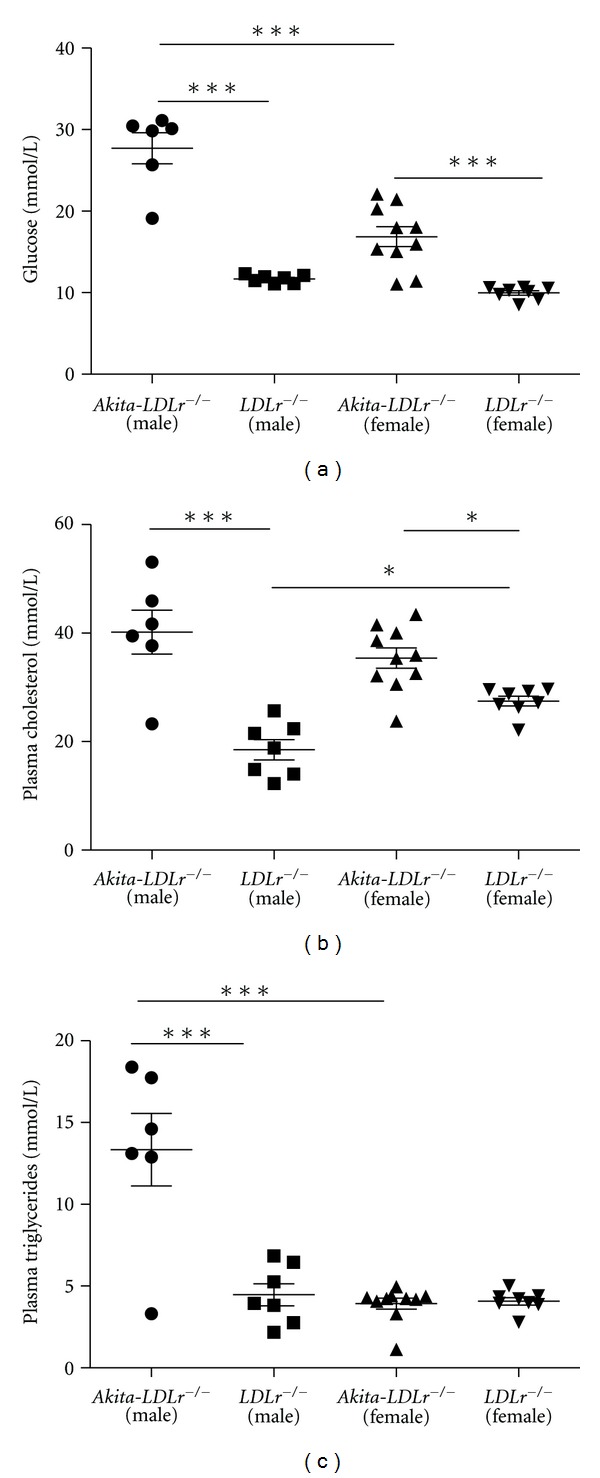
*Ak* *it* *a*-*LDLr* ^−/−^mice display gender-specific metabolic profiles. (a) Average blood glucose levels from measurements obtained between 9 and 24 weeks of age, (b) average plasma cholesterol, and (c) average plasma triglyceride levels of male and female *Akita*-*LDLr* ^−/−^ and *LDLr* ^−/−^ mice. Values are presented as individual mice and as mean ± SEM. Two-way ANOVA revealed interactions between diabetes and gender ((a)***, (b)**, (c)***), and significant effect of diabetes ((a)***, (b)***, (c)***) and of gender ((a)***, (c)***). Bonferroni post hoc test yielded *P < 0.05, ***P < 0.001.

*Ak* *it* *a*-*LDLr* ^−/−^ male mice displayed severe hyperglycemia with an average glucose level of 27.7 ± 4.6 mM, whereas *Akita*-*LDLr* ^−/−^ female mice had a milder phenotype with an average glucose of 16.9 ± 3.8 mM (Figure 1(a) and see Figure 1(a) in Supplementary Material available online at doi:10.1155/2012/176162). *LDLr* ^−/−^ mice were normoglycemic (11.7 ± 0.49 mM and 9.97 ± 0.76 mM; male and female, resp.). As it has been described for *Akita* mice [26], *Akita*-*LDLr* ^−/−^ males failed to gain as much weight as nondiabetic *LDLr* ^−/−^ males, and therefore had a lower body weight at 24 weeks of age (25.5 ± 1.1 g versus 31.6 ± 4.1 g, *P* < 0.01). No differences in body weight were observed between females (24.3 ± 1.9 g and 23.9 ± 2.9 g; diabetic and nondiabetic, resp.). Cholesterol levels in male *Akita*-*LDLr* ^−/−^ mice were increased two-fold compared to male *LDLr* ^−/−^ controls (40.1 ± 9.8 mM versus 18.3 ± 4.9 mM, *P* < 0.001; Figure 1(b)). Female *Akita*-*LDLr* ^−/−^ mice displayed a more modest increase in plasma cholesterol compared to female *LDLr* ^−/−^ controls (35.5 ± 6.0 mM versus 27.5 ± 2.5 mM, *P* < 0.01). Nondiabetic female *LDLr* ^−/−^ mice had elevated cholesterol levels when compared to nondiabetic male *LDLr* ^−/−^ mice (27.5 ± 2.5 mM versus 18.4 ± 4.9 mM, *P* < 0.05; Figure 1(b)). Moreover, *Akita*-*LDLr* ^−/−^ males, but not females, exhibited elevated triglyceride levels compared to their nondiabetic counterparts (Figure 1(c)).

### 3.2. Male *Akita-LDLr* ^−/−^ Mice Display Increased Atherosclerosis Compared to *LDLr* ^−/−^ Controls 

**Figure 2:**
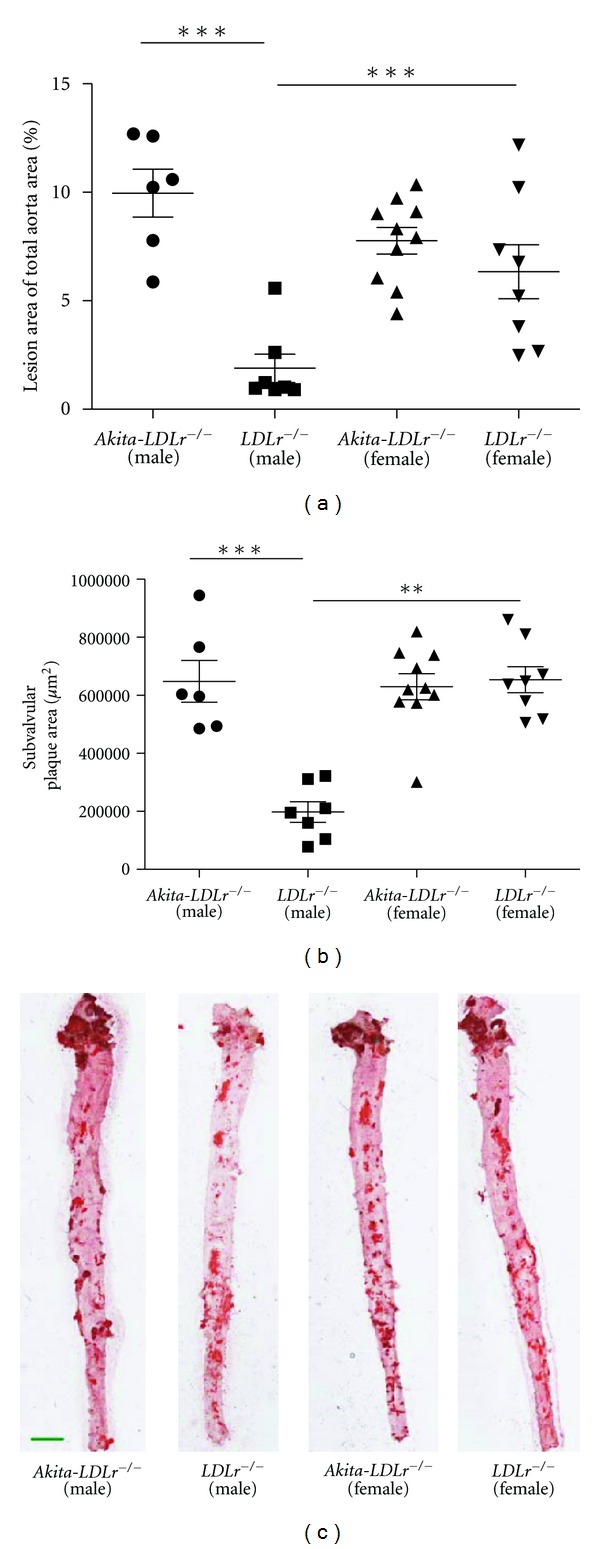
Male *Akita*-*LDLr* ^−/−^ mice have increased atherosclerosis compared to male *LDLr* ^−/−^ mice. Mice were sacrificed at 24 weeks of age and atherosclerosis were quantified both in *en face* preparations of the aorta (a) as well as in sections from the aortic root (b). Percentage plaque area of total vessel area in the aorta and representative *en face* preparations of the aortas stained with Oil red O (dark red-colored) are presented in (a) and subvalvular lesion areas are presented in (b). Values are presented as individual mice and as mean ± SEM. Two-way ANOVA revealed interactions between diabetes and gender ((a)**, (b)***), and significant effect of diabetes ((a)***, (b)***) and of gender (b)***. Bonferroni post hoc test yielded **P < 0.01, ***P < 0.001. Scale bar = 2 mm.

*En face* Oil red O staining of aortas of 24 weeks of age mice was performed in order to quantify atherosclerotic burden. Male *Akita*-*LDLr* ^−/−^ mice had a five-fold increase in lesion area compared to male *LDLr* ^−/−^ control mice (9.97% ± 2.70% versus 1.89% ± 1.74%, *P* < 0.001; Figure 2(a)). Nondiabetic female *LDLr* ^−/−^ were found to have a significantly larger lesion area than nondiabetic male *LDLr* ^−/−^ (6.34% ± 3.52% versus 1.89% ± 1.74%, *P* < 0.001; Figure 2(a)). However, diabetes had no significant effect on lesion area in the aorta of female mice (*Akita*-*LDLr* ^−/−^ versus *LDLr* ^−/−^; Figure 2(a)). In an analysis of the plaque area in the aortic arch separately, male *Akita*-*LDLr* ^−/−^ mice had a 5-fold increase in lesion area compared to male *LDLr* ^−/−^ mice (43.1% ± 11.7% versus 9.35% ± 6.68%, *P* < 0.001), whereas female *Akita*-*LDLr* ^−/−^ mice only displayed a trend toward a 1.4-fold increase in lesion area compared to female *LDLr* ^−/−^ (39.9% ± 7.7% versus 28.7% ± 14.5%, *P* = 0.05; unpaired *t*-test). When atherosclerosis was studied in the aortic root, the subvalvular lesion area was significantly increased in *Akita*-*LDLr* ^−/−^ male mice compared to nondiabetic *LDLr* ^−/−^ male mice (648,000 ± 177,000 *μ*m^2^ versus 198,000 ± 94,000 *μ*m^2^, *P* < 0.001, Figure 2(b)). As we found in the aorta, nondiabetic female *LDLr* ^−/−^ mice had significantly larger subvalvular plaque area than male *LDLr* ^−/−^ mice; however, diabetes had no further impact on plaque size in female mice (*Akita*-*LDLr* ^−/−^ versus *LDLr* ^−/−^, Figure 2(b)). 

### 3.3. Male *Akita-LDLr* ^−/−^ Mice Display Increased Accumulation of Inflammatory Cells in Atherosclerotic Lesions Compared to *LDLr* ^−/−^ Controls

**Figure 3:**
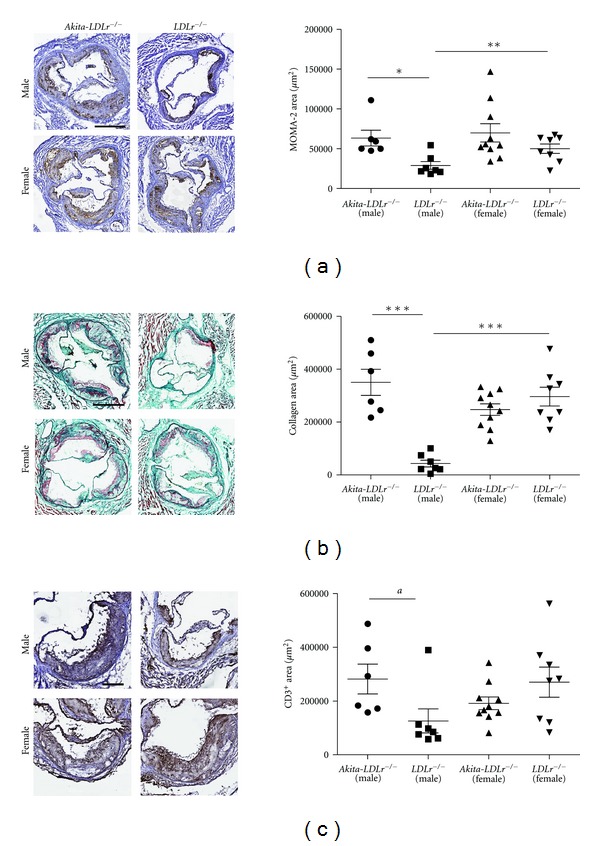
Male *Akita*-*LDLr* ^−/−^ mice have increased macrophages, T-lymphocytes, and collagen in subvalvular lesions compared to male *LDLr* ^−/−^ mice. Subvalvular lesions from 24-week-old mice were stained and quantified for monocytes/macrophages using MOMA-2 antibody (a), for collagen using Masson's trichrome staining (b), or for T-lymphocytes using anti-CD3 (c). Representative monocytes/macrophages, collagen and T-lymphocytes stainings are shown in (a)–(c). Values are presented as individual mice and as mean ± SEM. Two-way ANOVA revealed interactions between diabetes and gender ((b)*** and (c)*), and significant effect of diabetes ((a)** and (b)***) and of gender ((b)*). Bonferroni post hoc test yielded *P < 0.05, **P < 0.01, ***P < 0.001. *a*: *P* < 0.05; Mann-Whitney. Scale bar = 500 *μ*m in (a) and (b) and scale bar = 200 *μ*m in (c).

Monocyte and T-cell recruitment to the plaque plays a central role in atherogenesis. To investigate the effect of diabetes on monocyte/macrophage infiltration, we stained sections of the aortic root with MOMA-2. Subvalvular lesions of male *Akita*-*LDLr* ^−/−^ mice had larger areas infiltrated by macrophages as assessed by MOMA-2 immunoreactivity, compared to control *LDLr* ^−/−^ mice (63,000 ± 24,000 *μ*m^2^ versus 29,000 ± 13,000 *μ*m^2^, *P* < 0.05; Figure 3(a), see Figure 2 in Supplementary Material available online at doi: 10.1155/2012/176162). However, although having larger macrophage areas, the percentage of plaque area stained with MOMA-2 was lower in male *Akita*-*LDLr* ^−/−^ mice than in controls (9.8 ± 2.1% versus 17.6 ± 7.6%, *P* < 0.05). No statistically significant differences were observed regarding the female *Akita*-*LDLr* ^−/−^ mice versus control *LDLr* ^−/−^ mice. To address whether diabetes resulted in increased accumulation of T-cells in subvalvular atherosclerotic lesions, sections from the aortic root were stained with anti-CD3 and quantified. Male *Akita*-*LDLr* ^−/−^ mice had larger areas infiltrated by T-cells compared to control male *LDLr* ^−/−^ mice (28,000 ± 14,000 *μ*m^2^ versus 13,000 ± 12,000 *μ*m^2^, *P* < 0.05; Figure 3(c)). Since ABCA1-deficient macrophages display enhanced inflammatory responses [27], we stained and quantified subvalvular lesions for ABCA1 to see if the increased inflammation seen in male *Akita*-*LDLr* ^−/−^ mice could be explained by differences in ABCA1. *Akita*-*LDLr* ^−/−^ male mice had increased amount of ABCA1 in lesions compared to nondiabetic mice, whereas no such differences were observed in female mice (see Figure 3 in Supplementary Material available online at doi: 10.1155/2012/176162). Plaque from female *LDLr* ^−/−^ mice contained increased amount of ABCA1 compared to male *LDLr* ^−/−^ mice. The percentage of plaque area stained with anti-ABCA1 was decreased in male *Akita*-*LDLr* ^−/−^ mice (23% ± 9% versus 27% ± 3%, *P* = 0.05) compared to nondiabetic mice, but the same trend was seen in female mice (17% ± 5% versus 21% ± 3%, *P* = 0.05). Plaques from male *Akita*-*LDLr* ^−/−^ mice had significantly larger areas of collagen compared to male *LDLr* ^−/−^ mice (350,000 ± 121,000 *μ*m^2^ versus 43,000 ± 34,000 *μ*m^2^; *P* < 0.001), whereas there was no difference between *Akita*-*LDLr* ^−/−^ female mice and nondiabetic female *LDLr* ^−/−^ mice (Figure 3(b)). Again, gender differences were observed in nondiabetic *LDLr* ^−/−^ mice, with female mice having significantly larger collagen areas than male mice (Figure 3(b)). 

### 3.4. Effect of Diabetes on the Expression of Inflammatory Genes in the Brachiocephalic Artery

To determine if the increase in inflammatory cells in lesions of male *Akita*-*LDLr* ^−/−^ mice was preceded by inflammatory cytokines, we measured the expression of IL-1*β*, IL-6, VCAM, OPN, MMP-9, MCP-1, and MIP-2 in the brachiocephalic artery of 18 weeks of age *LDLr* ^−/−^ or *Akita*-*LDLr* ^−/−^ mice (Table 1). MIP-2 levels were increased in female *LDLr* ^−/−^ mice compared to female *Akita*-*LDLr* ^−/−^ mice, whereas there was no difference between diabetic and nondiabetic male mice. There was a tendency to increased MMP-9 levels in *Akita*-*LDLr* ^−/−^ mice compared to *LDLr* ^−/−^ mice including both genders (0.12 ± 0.05 versus 0.077 ± 0.05, *P* = 0.09), though this increase was more evident in female mice than in male mice. Moreover, in diabetic mice there were associations between plasma glucose levels and osteopontin expression (*r* = 0.80, *P* < 0.01), MCP-1 expression (*r* = 0.89, *P* < 0.001), or IL-6 expression (*r* = 0.61, *P* < 0.05), which were not conserved in nondiabetic mice. 

### 3.5. Effect of Diabetes on Myocardial Fibrosis

To determine if the increased atherosclerosis in *Akita*-*LDLr* ^−/−^ mice was associated with increased myocardial fibrosis, we measured collagen content in the muscular tissue of the heart, but we were only able to detect minor amounts of collagen in the tissue. Moreover, we did not find any differences between the groups (data not shown). In addition, we analyzed macrophage content in the heart muscle tissue, but did not find any signs of inflammation. 

### 3.6. Male *Akita-LDLr* ^−/−^ Mice Display Increased Levels of IL-1**β**

**Figure 4:**
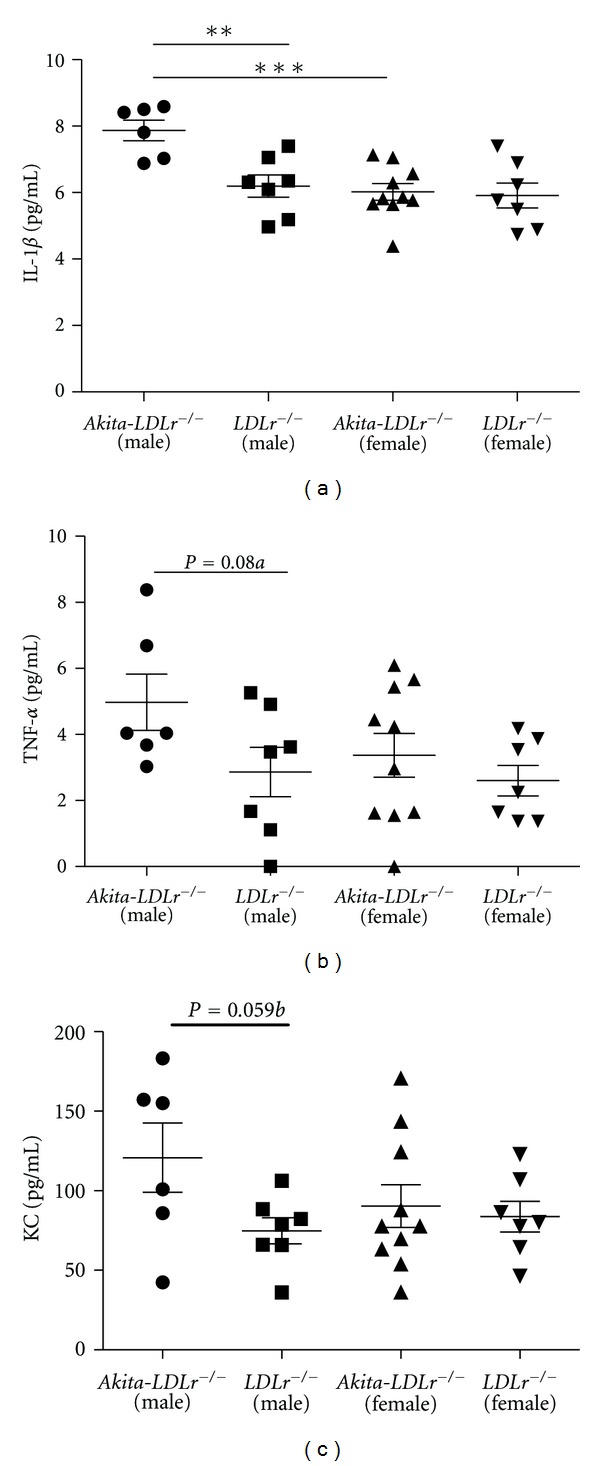
Male *Akita*-*LDLr* ^−/−^ mice have increased inflammatory cytokines in plasma. Plasma samples from 24-week-old *Akita*-*LDLr* ^−/−^ and *LDLr* ^−/−^ mice were analyzed for IL-1*β* (a), TNF*α* (b), or KC (c) using a multiplex assay. Values are presented as individual mice and as mean ± SEM. Two-way ANOVA revealed interactions between diabetes and gender ((a)*), and significant effect of diabetes ((a)*) and of gender ((a)**). Bonferroni post hoc test yielded **P < 0.01, ***P < 0.001. *a* and *b*: unpaired *t*-test.

Plasma levels of the proinflammatory cytokine IL-1**β** were significantly elevated in 24-week-old *Akita*-*LDLr* ^−/−^ males compared to *LDLr* ^−/−^ males of the same age (Figure 4(a)). Moreover, both TNF-*α* (*P* = 0.089; Figure 4(b)) and the neutrophil activating chemokine KC (CXCL1; *P* = 0.059; Figure 4(c)) displayed trends towards being increased in male *Akita*-*LDLr* ^−/−^ mice compared to male *LDLr* ^−/−^ control mice. In a linear regression analysis model, IL-1*β* was associated with average glucose levels in diabetic male and female mice, but not with cholesterol levels. There were no significant changes in plasma levels of IFN*γ*, IL-2, IL-4, IL-5, or IL-10 between the groups (Table 2). Total IL-12, including both anti-inflammatory IL-12p40 and proinflammatory IL-12p70, was decreased in male *Akita*-*LDLr* ^−/−^ mice compared to male *LDLr* ^−/−^ control mice (Table 2).

**Figure 5:**
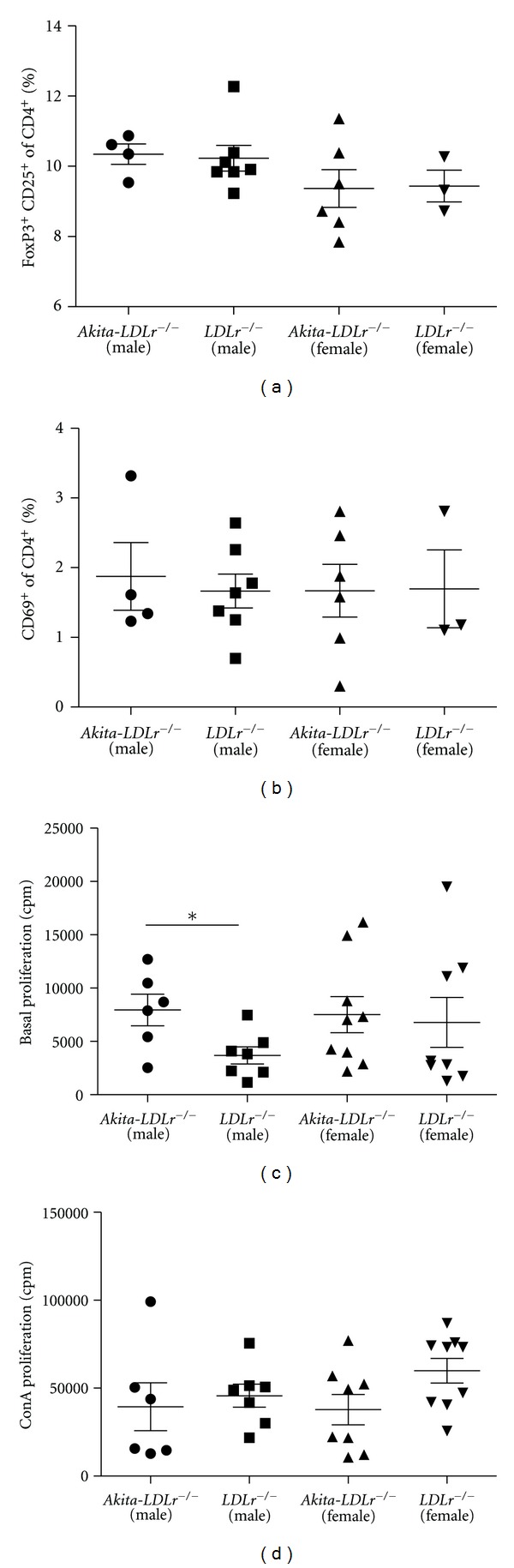
Diabetic *Akita*-*LDLr* ^−/−^ mice and nondiabetic *LDLr* ^−/−^ mice have similar levels of regulatory T-cells. Splenocytes from 24-week-old *Akita*-*LDLr* ^−/−^ and *LDLr* ^−/−^ mice were analysed for regulatory (CD4+CD25+FoxP3+) T-cells (a), activated (CD4+CD69+) T-cells (b) using flow cytometry or basal (c), and ConA stimulated (d) proliferation. Values are presented as individual mice and as mean ± SEM. *P < 0.05, Mann-Whitney test.

T-cell subsets have been shown to greatly influence the development of experimental atherosclerosis in mice [28]. To assess whether the increased plaque development reflected a change in the balance between activated conventional T-cells and anti-inflammatory regulatory T-cells, we performed flow cytometry on isolated splenocytes from 24-week-old mice. No differences in percentages of regulatory T-cells (CD4+CD25+FoxP3+, Figure 5(a)) or activated T-cells (CD4+CD69+, Figure 5(b)) were observed between the groups. To further determine the T-cell activation status, we measured basal and Concanavalin A- (ConA-) induced proliferation. Male *Akita*-*LDLr* ^−/−^ mice displayed higher basal proliferation compared to male *LDLr* ^−/−^ mice, whereas there was no difference in ConA stimulated proliferation (Figures 5(c) and 5(d)). No significant difference was observed between female mice.

## 4. Discussion

In this study, we characterize the *Akita*-*LDLr* ^−/−^ mouse as a model of diabetic atherosclerosis. We also investigate if the atherosclerotic disease in *Akita*-*LDLr* ^−/−^ mice is accompanied by increased inflammation in atherosclerotic lesions. We show that the *Akita*-*LDLr* ^−/−^ mouse has considerable gender differences with regard to metabolic profile, atherosclerotic disease, and inflammatory cells in atherosclerotic lesions. While male *Akita*-*LDLr* ^−/−^ mice exhibit severe hyperglycemia, hypercholesterolemia, and hypertriglyceridemia compared to nondiabetic *LDLr* ^−/−^ mice, female *Akita*-*LDLr* ^−/−^ mice show marked hyperglycemia, but only a modest increase in cholesterol levels and no changes in plasma triglycerides. Male *Akita*-*LDLr* ^−/−^ mice have a five-fold increase in aortic lesion area, a three-fold increase in subvalvular lesion area, and increased areas of macrophage and T-cell infiltration in the lesions compared to male *LDLr* ^−/−^ mice, but no such differences were seen in female mice. 

Estrogen and its receptors are regulators of glucose and lipid metabolism and rodent studies link estrogen to anti diabetic effects. Estrogen is also known to exert antiinflammatory effects in both humans and rodents [29]. It is possible that the gender differences reported in our study at least partly are due to the effects of estrogen, for example, plasma IL-1*β* levels are increased in male *Akita*-*LDLr* ^−/−^ mice compared to female *Akita*-*LDLr* ^−/−^ mice (Figure 4(a)). The reason why female *Akita*-*LDLr* ^−/−^ mice, despite induction of hyperglycemia, have no or only a minor increase in atherosclerosis, macrophage, and T-cell infiltration compared to female *LDLr* ^−/−^ mice could be due to the anti-inflammatory effects of estrogen.In this respect, it is important to note that hyperglycemia is less pronounced in females than in males (16.9 ± 3.8 mM versus 27.7 ± 4.6 mM). It is also important to note that nondiabetic female *LDLr* ^−/−^ mice have a 1.5-fold increase in cholesterol levels and 3-fold increase in subvalvular lesion area compared to nondiabetic male *LDLr* ^−/−^ mice. In fact, female nondiabetic *LDLr* ^−/−^ mice have the same subvalvular lesion area as male diabetic *Akita*-*LDLr* ^−/−^ mice, but the lesion area in female mice is not further increased in response to hyperglycemia. It is possible that the already high cholesterol levels in female nondiabetic *LDLr* ^−/−^ mice mask the effect of hyperglycemia on lesion formation in female diabetic *Akita*-*LDLr* ^−/−^ mice. Such observations have been made in other mouse models of diabetic atherosclerosis. For example, Reaven et al. studied male *LDLr* ^−/−^ mice on a high fat diet, which were made diabetic using streptozotocin [30]. In that study, diabetic and nondiabetic mice had similar plasma cholesterol levels (25.1 versus 25.9 mM); however, diabetic mice had increased glucose levels, increased triglyceride levels, and increased formation of AGE epitopes in the artery wall. Despite this, there were no differences in atherosclerosis between diabetic and nondiabetic mice. The authors suggest that under conditions of marked hypercholesterolemia, there is no effect of hyperglycemia and/or of enhanced AGE formation on atherogenesis in *LDLr* ^−/−^ mice. Thus the observed gender differences in atherosclerotic lesions and inflammation in our study may have two possible explanations: (1) the already high cholesterol levels in female nondiabetic *LDLr* ^−/−^ mice could mask the effect of hyperglycemia on lesion formation in female diabetic *Akita*-*LDLr* ^−/−^ mice, and (2) the less pronounced hyperglycemia in female *Akita*-*LDLr* ^−/−^ mice compared to male *Akita*-*LDLr* ^−/−^ mice may result in less atherosclerotic disease and less inflammation.

Our results partly confirm a recent study of LDLr-deficient *Akita* mice published by Zhou et al. [18]. Zhou et al. reported hyperglycemia and hyperlipidemia accompanied by increased atherosclerotic disease in male and female *Akita*-*LDLr* ^−/−^ mice. In that study, however, they found no difference in blood glucose levels between male and female mice (23.5 ± 9 mM versus 21.8 ± 7.2 mM), whereas we found that male *Akita*-*LDLr* ^−/−^ mice had higher glucose levels than females (27.7 ± 4.6 mM versus 16.9 ± 3.8 mM, *P* < 0.001). High-fat diet induces insulin resistance and diabetes in C57/Bl6 mice [31] and there is gender difference [32, 33]. However, it has also been reported that there is a large difference in glucose levels in male versus female *Akita* mice, even on a low-fat diet (27.3 ± 5.3 mM in males versus 13.6 ± 3.8 mM in females) [26], and the authors speculate that estrogen and prolactin play a protective role in the females. The levels reported by Yoshioka et al. are similar to the levels we report (27.7 ± 4.6 mM in males versus 16.9 ± 3.8 mM in females). In addition, whereas we found no significant difference in lesion area in female mice, neither in *en face* preparations of the aorta nor in subvalvular lesions, Zhou et al. reported a significant increase in subvalvular lesions of female *Akita*-*LDLr* ^−/−^ mice compared to female *LDLr* ^−/−^ mice. However, in agreement with our study, the increase in lesion area in diabetic mice compared to nondiabetic mice were larger in males (224%) than in females (30%). The mice in our study were fed a high fat diet containing 0.15% cholesterol, whereas Zhou et al. fed the mice a low-fat diet with 0.02% cholesterol, thus there was a considerable difference in the size of the subvalvular lesion area in our study compared to Zhou et al. (630000 *μ*m^2^ versus approx. 350000 *μ*m^2^, resp., for female diabetic mice). The difference in size of subvalvular lesions may also explain why Zhou et al. report significant differences in diabetic female *Akita*-*LDLr* ^−/−^ mice compared to nondiabetic *LDLr* ^−/−^ mice. The subvalvular lesions in our study, which is almost the double size compared to the ones reported by Zhou et al., may be at a later atherosclerotic stage, at which the differences in female mice may be evened. Thus, since the subvalvular lesions are one of the earliest atherosclerotic lesions in mice, there may be differences in lesion size at other locations. This is supported by our finding that female *Akita*-*LDLr* ^−/−^ mice displayed a trend towards larger lesion area in the aortic arch compared to female *LDLr* ^−/−^ mice. 

Further, Zhou et al. determined liver specific expression of genes involved in lipid metabolism and inflammation by quantitative PCR [18]. In a recent report Jun et al. showed increased atherosclerosis in male apoE-deficient *Akita* mice, but did not analyze female mice [17]. In the latter study, the authors analyzed expression of lipoprotein receptors in the liver, lipid secretion from the liver and, plasma lipid profile. Thus, both these recently published studies have focused on lipid metabolism and thus mainly studied differences in the liver. To assess whether the increased atherosclerosis seen in diabetic mice is accompanied by increased inflammation in atherosclerotic vessels, we characterized and quantified inflammatory cells in subvalvular lesions by immunohistochemistry. In addition, we used quantitative PCR to analyze the expression of inflammatory genes in the brachiocephalic artery, which is one of the most plaque prominent locations in the arterial tree in mice. 

Studies of plaques derived from both type 1 and 2 diabetes patients have shown increased accumulation of T-cells and macrophages [34]. Similar to humans, subvalvular lesions of *Akita*-*LDLr* ^−/−^ male mice were enriched in both T-cells and macrophages compared to *LDLr* ^−/−^ controls. Surprisingly, *LDLr* ^−/−^ mice have a higher percentage of macrophages in the lesions than diabetic *Akita*-*LDLr* ^−/−^ mice, which probably reflects that the lesions are at an earlier stage, whereas more advanced lesions display increased infiltration of smooth muscle cells and more fibrous tissue. This is supported by increased collagen content in *Akita*-*LDLr* ^−/−^ male mice compared to controls.

It was previously shown that *Akita*-*LDLR* ^−/−^ mice displayed decreased mRNA levels in the liver of several genes involved in lipid metabolism (Srebp1a, Srebp1c, AbcA1, Lxr*α*, and Cyp7b1) [18]. Some of these genes have been shown to modulate atherosclerotic disease. For example, since LXRs promote cholesterol efflux via upregulation of the ABC family [35], one would expect that LXRs would have antiatherogenic properties due to increased reverse cholesterol transport in the aorta. Indeed, LXR*αβ*-deficient macrophages displayed increased accumulation of cholesterol [36], and apoE- and LXR*α*-deficient mice show increased atherosclerotic disease [37, 38]. In agreement with this, treatment with LXR agonist reduces atherosclerosis in mice [39–42]. However, LXRs do not only affect atherosclerosis due to increased reverse cholesterol transport. It has been shown that LXRs are negative regulators of macrophage inflammatory gene expression. *In vitro* studies show that LXR ligands inhibit the expression of inflammatory genes such as *IL*-6, *IL*-*β*, *MCP*-1, *MMP*-9, and osteopontin [43–45]. Moreover, *in vivo* treatment of *apoE* ^−/−^ mice with LXR agonists resulted in substantially reduced *MMP*-9 gene expression in the aortas [44]. In our study, quantitative gene expression analysis of either *IL*-6, *IL*-1*β*, *MCP*-1, or osteopontin in the brachiocephalic artery did not reveal any difference between *Akita*-*LDLr* ^−/−^ or control mice. There was a tendency to increased MMP-9 gene expression in *Akita*-*LDLr* ^−/−^mice compared to *LDLr* ^−/−^mice including both genders, though this increase was more evident in female mice than in male mice. Also ABCA1 has been reported to have anti-inflammatory properties shown by ABCA1-deficient macrophages, which displayed increased TNF*α* expression upon LPS stimulation [27]. Moreover, bone marrow transplantations demonstrated an antiatherogenic function of ABCA1 in macrophages independently of changes in plasma lipids [46]. In our study, total ABCA1 levels, analyzed by immunohistochemistry, were increased in subvalvular lesions of male *Akita*-*LDLr* ^−/−^mice. Although the percentage of plaque area stained with anti-ABCA1 was decreased in male *Akita*-*LDLr* ^−/−^ mice, the same difference was present in female mice. In conclusion, in our study we find no evidence suggesting that downregulation of *AbcA*1 or *Lxr*α** gene in lesions could explain the gender difference in inflammatory cells observed in subvalvular lesions of *Akita*-*LDLr* ^−/−^ mice. On the other hand, Zhou et al. reported increased TNF*α*, MCP-1, and IL-1*β* staining in lesions of *Akita*-*LDLr* ^−/−^ mice compared to *LDLr* ^−/−^ mice. Differences in our study compared to the study by Zhou et al., for example, the diet, location of the lesions, or protein versus mRNA analysis, may explain the reported differences in cytokines in atherosclerotic lesions.

Diabetes is a strong independent risk factor for heart failure [47, 48]. In a previous paper Basu et al. found that normolipidemic *Akita* C57Bl6 mice were characterized by diastolic dysfunction at three and six months of age, but preserved systolic function [49]. Moreover, there was no evidence of myocardial hypertrophy or fibrosis in the diabetic mice. The latter is in agreement with our data, showing no or only minor collagen content in cardiac tissue and no differences between diabetic and nondiabetic mice.

Several papers have shown that *Akita* male mice (on normolipidemic background C57bl6 background) develop elevated systolic blood pressure [50–52], which is not present in female mice [50]. Basu et al. measured heart rate in *Akita* C57BL/6J mice, but did not find any differences between diabetic and nondiabetic mice [49]. In humans, increased blood pressure is a risk factor for the development of atherosclerotic disease; however, in mice the relationship between blood pressure and atherosclerosis is less clear. While several reports have demonstrated reduced atherosclerosis in mice with decreased blood pressure, other studies have shown that changes in blood pressure do not affect atherosclerotic disease (reviewed in [53]). Thus, it is difficult to predict whether differences in blood pressure could underlie the gender differences in atherosclerosis observed in *Akita*-*LDLr* ^−/−^ mice. 

It is widely recognized that T-cells influence atherosclerosis in mice [28]. For example, regulatory T-cells have in several studies been shown to protect against atherosclerosis [54]. Since we did not find any differences in percentages in either the protective regulatory (CD4+CD25+FoxP3+) T-cells or the putatively harmful activated (CD4+CD69+) T-cells, we suggest that diabetic atherosclerosis in these mice is not induced by an overall immune activation. 

## 5. Conclusion

In conclusion, both male and female *Akita*-*LDLr* ^−/−^ mice are hyperglycemic compared to control *LDLr* ^−/−^ mice. However, whereas male *Akita*-*LDLr* ^−/−^ mice have a 2-fold increase in plasma cholesterol and 3-fold increase in triglyceride levels, female *Akita*-*LDLr* ^−/−^ mice have only a modest diabetes-induced increase in cholesterol and no increase in triglyceride levels. This is accompanied by a dramatic increase in atherosclerosis as well as increased plaque inflammation in male *Akita*-*LDLr* ^−/−^ mice, but no significant changes in plaque size or inflammatory cells in lesions in female *Akita*-*LDLr* ^−/−^ mice compared to nondiabetic *LDLr* ^−/−^ mice. We propose that the *Akita*-*LDLr* ^−/−^ mouse is a promising tool for studying development of cardiovascular disease both in a setting of severe as well as a more moderate increase in cholesterol levels. 

## Supplementary Material

Supplementary fig.1: Blood glucose (a) and plasma cholesterol (b) levels in *Akita-LDLr^−/−^* and *LDLr^−/−^* male and female mice. Values represent mean ± SEM.
Supplementary fig.2: Macrophage staining of subvalvular lesions (high magnification). The positive staining (brown) reflects both the presence of macrophages in the plaque and the presence of cell debris from necrotic macrophages in the core of the lesion. Scale bar=100 *μ*m.
Supplementary fig.3: Male *Akita-LDLr^−/−^* mice have increased ABCA1 levels in subvalvular lesions compared to male *LDLr^−/−^* mice. Subvalvular lesions from 24-week old mice were stained and quantified for ABCA1. Values are presented as individual mice and as mean ± SEM. Two-way ANOVA revealed interactions between diabetes and gender (∗∗∗), and significant effect of diabetes (∗∗∗) and of gender (∗). Bonferroni post hoc test yielded ∗ P<0.05, ∗∗∗ P<0.001.

## Figures and Tables

**Table 1:** Expression of inflammatory genes in the brachiocephalic artery of 18-week-old *Akita*-*LDLr* ^−/−^ mice and *LDLr* ^−/−^ mice.

	IL-1*β*	OPN	IL-6	VCAM	MMP-9	MCP-1	MIP2
Males							
*Ak* *it* *a*-*LDLr* ^−/−^ (*n* = 6)	0.11 ± 0.07	4.2 ± 7.2	0.44 ± 0.90	5.5 ± 3.7	0.14 ± 0.05	0.22 ± 0.26	0.050 ± 0.04
*LD* *Lr* ^−/−^ (*n* = 5)	0.09 ± 0.12	3.4 ± 6.6	1.1 ± 2.1	6.8 ± 4.1	0.10 ± 0.05	0.20 ± 0.24	0.059 ± 0.05
Females							
*Ak* *it* *a*-*LDLr* ^−/−^ (*n* = 5)	0.06 ± 0.02	3.5 ± 3.2	0.11 ± 0.11	9.4 ± 8.6	0.09 ± 0.04	0.18 ± 0.08	0.04 ± 0.01*
*LD* *Lr* ^−/−^ (*n* = 4)	0.08 ± 0.04	4.7 ± 2.3	0.29 ± 0.23	10.0±8.1	0.05 ± 0.02	0.25 ± 0.14	0.06 ± 0.02

*P < 0.05 versus *LDLr* ^−/−^ females; Mann-Whitney.

Levels of mRNA were measured by real-time RT-PCR and were normalized to the expression of the housekeeping control gene cyclophilin B. values represent mean ± SD.

**Table 2:** Plasma cytokine levels in *kita*-*LDLr* ^−/−^ mice and control *LDLr* ^−/−^ mice at 24 weeks of age.

	IFN*γ*	IL-2	IL-4	IL-5	IL-10	IL-12 (p40 + p70)
Males						
*Ak* *it* *a*-*LDLr* ^−/−^ (*n* = 5-6)	2.0 ± 0.71	21.2 ± 3.2	3.0 ± 1.2	9.7 ± 1.1	104 ± 15.2	978 ± 139*
*LD* *Lr* ^−/−^ (*n* = 7)	2.1 ± 0.87	20.1 ± 3.2	3.0 ± 1.2	9.6 ± 1.7	90.4 ± 19.4	1410 ± 316
Females						
*Ak* *it* *a*-*LDLr* ^−/−^ (*n* = 10)	6.3 ± 7.8	17.9 ± 1.5	12.0 ± 15.9	17.0 ± 8.5	218 ± 229	1290 ± 304
*LD* *Lr* ^−/−^ (*n* = 7)	10.8 ± 20.1	16.3 ± 3.6	12.4 ± 23.3	14.3 ± 7.4	198 ± 203	1140 ± 305

*P < 0.05 versus *LDLr* ^−/−^ males; Bonferroni post-hoc test.

Two-way ANOVA revealed interactions between diabetes and gender for IL-12 (*P* < 0.05), and significant effect of gender for IL-5 (*P* < 0.05).

Values represent mean ± SD.
