# Supplementary figures and images for: Increased Inflammation in Atherosclerotic Lesions of Diabetic Akita-LDLr −/− Mice Compared to Nondiabetic LDLr −/− Mice
Source: Exp Diabetes Res. 2012 Nov 28;2012:176162. doi: 10.1155/2012/176162 (PMC3515907; doi:10.1155/2012/176162)

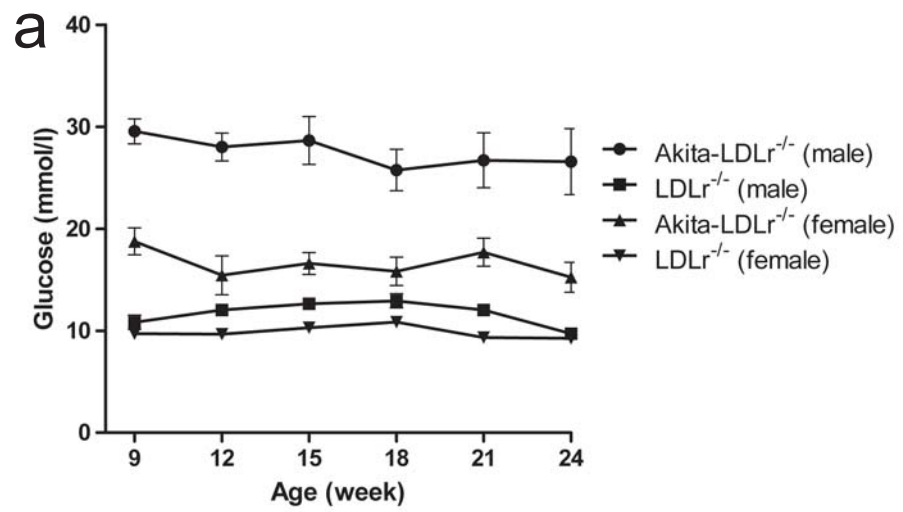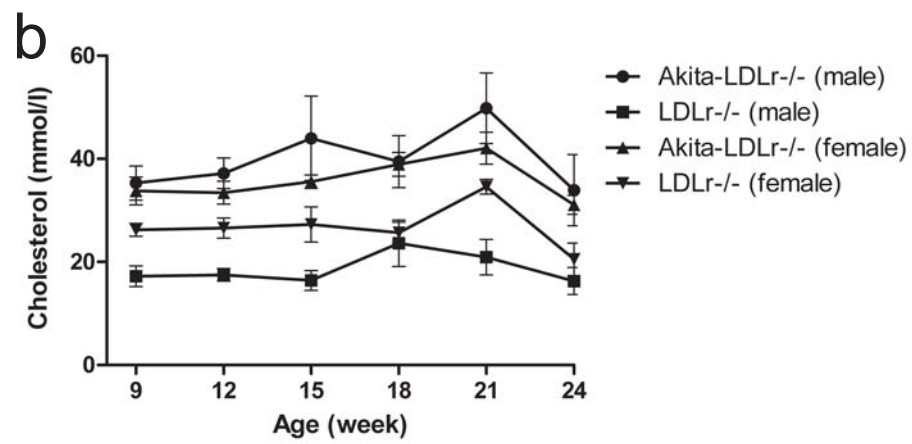

Supplement: Supplementary file 1 — Supplementary fig.1: Blood glucose (a) and plasma cholesterol (b) levels in Akita-LDLr−/− and LDLr−/− male and female mice. Values represent mean ± SEM. Supplementary fig.2: Macrophage staining of subvalvular lesions (high magnification). The positive staining (brown) reflects both the presence of macrophages in the plaque and the presence of cell debris from necrotic macrophages in the core of the lesion. Scale bar=100 μm. Supplementary fig.3: Male Akita-LDLr−/− mice have increased ABCA1 levels in subvalvular lesions compared to male LDLr−/− mice. Subvalvular lesions from 24-week old mice were stained and quantified for ABCA1. Values are presented as individual mice and as mean ± SEM. Two-way ANOVA revealed interactions between diabetes and gender (∗∗∗), and significant effect of diabetes (∗∗∗) and of gender (∗). Bonferroni post hoc test yielded ∗ P<0.05, ∗∗∗ P<0.001. [file 176162.f1.pdf]

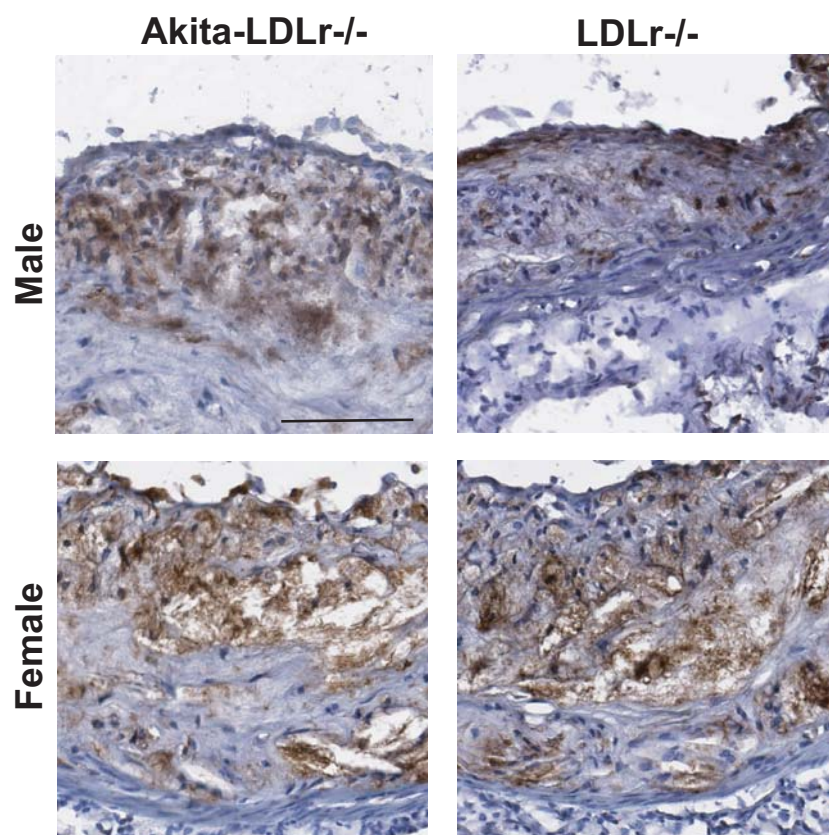

Supplement: Supplementary file 2 [file 176162.f2.pdf]

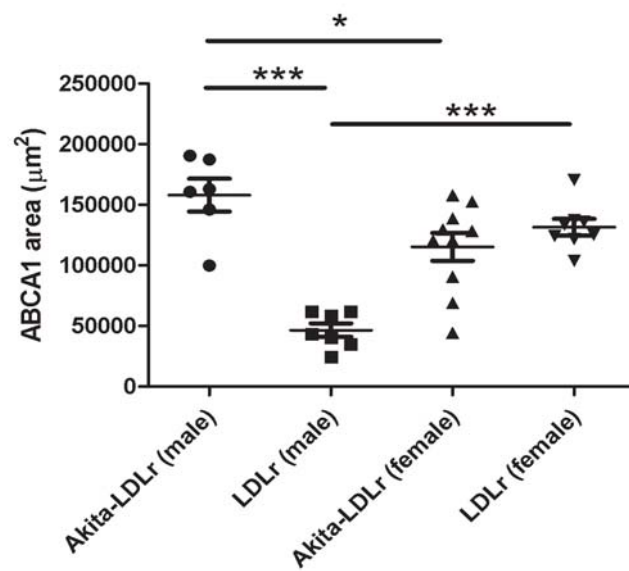

Supplement: Supplementary file 3 [file 176162.f3.pdf]
